# Minimally invasive techniques: full endoscopic inferior parietal lobule approach for an endoventricular removal of a left trigone meningioma grade I

**DOI:** 10.3171/2023.1.FOCVID2296

**Published:** 2023-04-01

**Authors:** Clarissa A. E. Gelmi, Giulio Cecchini, Giovanni Vitale, Francesco Di Biase

**Affiliations:** 1 Neurosurgery Unit, Regional Hospital San Carlo, Potenza, Italy; and; 2 Neurosurgery Unit, Translational Medicine Department, University of Ferrara, Italy

**Keywords:** minimally invasive neurosurgery, intraventricular meningioma, fully endoscopic

## Abstract

Intraventricular meningiomas are rare tumors, mainly localized in the lateral ventricle trigone. Several microscopic surgical approaches have been proposed according to the location of the tumors in the atrium. The authors present the case of a 68-year-old woman who had an increscent medically resistant headache with a left trigone meningioma. In the video, they demonstrate the four-hands surgical technique through a minimally invasive fully endoscopic retractor-less transcortical approach. They also discuss the background of these tumors and the technical nuances of the procedure, including the differences between an endoscopic surgical and a microsurgical one.

The video can be found here: https://stream.cadmore.media/r10.3171/2023.1.FOCVID2296

## Transcript

### 0:25 Clinical Presentation.

We presented the case of a 68-year-old woman who suffered of a persistent headache resistant to medical therapy. She underwent an MRI, which showed the presence of an intraventricular lesion in the left trigone, with the main suspicion of a meningioma.

### 0:48 Rational for Surgery and Eventual Alternatives.

According to the size of the tumor and the symptoms’ onset, surgery was indicated to avoid an acute hydrocephalus, intracranial hypertension, or development of neurological deficits. The risks of this procedure include eventual damages at the optic radiation, language areas, motor deficits, and Gerstmann’s syndrome for the subcortical parietal areas. Gamma Knife and radiosurgery are rarely indicated except for patients who can’t undergo general anesthesia or refuse surgery.

To avoid damages at near-eloquent areas, we performed a full endoscopic inferior parietal lobule transcortical approach with a lateromedial/superoinferior direction, based on the preoperative tractography.

### 1:25 Surgical Setup.

The patient was placed in a lateral position with the head turned three-quarters on the right side, fixed with Mayfield head holder. The equipment was made by the surgical endoscope, microsurgical tubular instruments, ultrasound aspirator, and neuronavigator, which allowed us to study the surgical trajectory.

### 1:44 Four-Hands Surgical Technique.

Four-hands surgical technique was performed, consisting in two surgeons as first operators, of which in this case one holds the endoscope and spatula, the other one the surgical instruments, such as US aspirator, suction, bipolar, dissector, and microscissor. Both hands of the surgeons move in a coordinated way to guarantee a complete surgical vision by reducing the dynamic retraction.

### 2:08 Full Endoscopic Transcortical Approach.

A minimally invasive linear skin incision was performed centered on the mini-craniotomy, followed by the corticectomy. A retractor-less transcortical approach was made with the only use of a spatula and dissector, to make gentle dynamic retraction of the interested parenchyma to avoid iatrogenic cerebral contusions.

Considering the minimally invasive approach and the surgical site, it’s important to first debulk the lesion to easily find then its pedicle. Therefore, a mild debulking was performed through ultrasound aspirator. Usually, these lesions are characterized by a dura-like layer on the surface, which we could appreciate with some adherences between the ependyma and the tumor, already identified through the preoperative MRI, which have been gently detached.

A first pedicle of the lesion was then seen, attached to the choroidal plexus in its cranial aspect, facing the anterior part of atrium, toward the middle sella and more anteriorly to the frontal horn. The vascular pedicle of the tumor should be identified and coagulated at the earliest possible time to avoid excessive bleeding from the ependymal surface, which can be challenging to control. We used bipolar forceps to coagulate and then cut it with microscissors to initially mobilize the tumor.

After having partially moved the lesion, we could find the choroidal plexus on the occipital side, going downward to the temporal horn with the lateral-posterior left choroidal artery. Thanks to the endoscope surgical view, we could also appreciate the column of fornix in white, which turned around the thalamus on the anteromedial aspect.

After having detached the lesion from the ependyma and rolling it on the surgical site, avoiding any retraction, we could appreciate the choroidal plexus still kept the lesion adherent to this area. This kind of surgical vision was possible only thanks to the endoscope.

We continued then to debulk the tumor to better expose its base of implant, which we finally cauterized, avoiding the rupture of the small cicatricial synechiae and choroidal vessels associated; otherwise, there may be small bleedings, which, although minimal, could be compromised the ventricular system. The choroidal plexus was completely cauterized and cut with microscissor.

We moved finally on the parieto-occipital portion of the choroidal plexus to completely detach the meningioma. An exploration excluded any other portion of attachment; the lesion was rolled in the surgical cavity to allow the surgeon to see if beyond the lesion any vessels were attached. Then we gently removed the tumor, appreciating the corticectomy was smaller than the lesion itself.

### 4:57 Background Disease.

Intraventricular meningiomas are rare tumors, mainly located in the trigone of the lateral ventricle.^1^ They usually origin from the meningothelial inclusion bodies in the tela choroidea or the mesenchymal stroma of choroid plexus,^2^ often surrounded by a dura-like layer, as seen in this case. Because of their size they usually become symptomatic due to block the CSF and their proximity to eloquent areas.

Surgery is indicated when symptomatic, and in literature are described different approaches according to the tumor position in the trigone and to avoid the nearby white matter fiber tracts with a microscopic view.^3,4^ The endoscopic approach gives a wider viewing range compared with the microscope and may reduce damage to normal brain tissue, facilitating total resection of the lesion and improving the surgical outcome.^5^ Surgical trajectory is based on the trigone tumor’s relationship with near white matter fiber tracts involved in the vision, language, motor functions, and integration of information.^6^

### 6:00 Postoperative Outcomes.

A postoperative CT scan excluded any complications. The patient underwent a postoperative MRI within 48 hours from surgery, which confirmed the gross-total resection of the lesion. She was discharged in her 6th postoperative day without neurological deficits.

She repeated the MRI during the follow-up, 2 months after surgery. The patient was in perfect condition without headache. The final diagnosis was grade I meningioma, according to the WHO classification.

## Acknowledgments

We are extremely grateful to Dr. Valeria Coppola, Neuroradiologist at Regional Hospital San Carlo, for her passion and extreme accuracy in helping us with the preoperative planning, creating specific tractography.

## Disclosures

The authors report no conflict of interest concerning the materials or methods used in this study or the findings specified in this publication.

## Author Contributions

Primary surgeon: Cecchini. Assistant surgeon: Di Biase. Editing and drafting the video and abstract: Gelmi. Critically revising the work: Gelmi, Cecchini, Vitale. Reviewed submitted version of the work: Gelmi, Cecchini. Approved the final version of the work on behalf of all authors: Gelmi. Supervision: Cecchini, Vitale, Di Biase.

## Supplemental Information

### Patient Informed Consent

The necessary patient informed consent was obtained in this study.

## References

[1] Choi JW Jung S Jung TY Jang WY Moon KS Kim IY Modified trans-middle temporal gyrus approach for trigonal tumor to preserve visual field J Korean Neurosurg Soc 2011 50 6 538 541 22323945 10.3340/jkns.2011.50.6.538 PMC3272519

[2] Nayar VV DeMonte F Yoshor D Blacklock JB Sawaya R Surgical approaches to meningiomas of the lateral ventricles Clin Neurol Neurosurg 2010 112 5 400 405 20197209 10.1016/j.clineuro.2010.02.005

[3] Timurkaynak E Rhoton AL Jr Barry M Microsurgical anatomy and operative approaches to the lateral ventricles Neurosurgery 1986 19 5 685 723 3785618 10.1227/00006123-198611000-00001

[4] Andrews JP Arora T Theodosopoulos P Berger MS Paramedian transparietal approach to a dominant hemisphere intraventricular meningioma: illustrative case J Neurosurg Case Lessons 2021 2 7 CASE21292 35855414 10.3171/CASE21292 PMC9265169

[5] Wang X Yang L Zhang H Yan Z She L Microsurgical and endoscopic posterior transcortical keyhole approach to the atrium of the lateral ventricle: a cadaveric study J Neurol Surg A Cent Eur Neurosurg 2015 76 4 261 267 25594819 10.1055/s-0034-1393928

[6] Stippich C Clinical Functional MRI: Presurgical Functional Neuroimaging 2nd ed. Springer 2015

